# Microscope-Integrated Augmented Reality-Assisted Navigation for C1–C2 Instrumentation: A Retrospective Single-Center Case Series

**DOI:** 10.3390/jcm15207812

**Published:** 2026-10-10

**Authors:** Chi-Ruei Li, Song-Ru Jhan, Hsi-Kai Tsou, Yu-Jui Chang, Mao-Shih Lin, Chung-Yuh Tzeng, Ting-Hsien Kao, Chiu-Chun Chen, Jheng-Yan Chen, Chih-Wei Huang

**Affiliations:** 1 Department of Neurosurgery, Neurological Institute, Taichung Veterans General Hospital, Taichung 407219, Taiwan; fantastic1694@gmail.com (C.-R.L.); mynewcellphoneyo@gmail.com (Y.-J.C.); ken70218@gmail.com (M.-S.L.); joshuakth@gmail.com (T.-H.K.); a5880018@gmail.com (C.-C.C.); abctom789@yahoo.com.tw (J.-Y.C.); 2 Institute of Medicine, Chung Shan Medical University, Taichung 40201, Taiwan; 3 Department of Radiology, Taichung Veterans General Hospital, Taichung 407219, Taiwan; song.ru.jhan@gmail.com; 4 Department of Post-Baccalaureate Medicine, College of Medicine, National Chung Hsing University, Taichung 402202, Taiwan; 5 College of Health, National Taichung University of Science and Technology, Taichung 404336, Taiwan; 6 Department of Orthopedics, Taichung Veterans General Hospital, Taichung 407219, Taiwan; tcy0545@gmail.com; 7 Institute of Biomedical Sciences, National Chung Hsing University, Taichung 402202, Taiwan; 8 Department of Rehabilitation, Jen-Teh Junior College of Medicine, Nursing and Management, Houlong, Miaoli County 356006, Taiwan

**Keywords:** augmented reality (AR), atlantoaxial joint, atlantoaxial joint/surgery, case series, image-guided surgery, joint instability, microscopic surgery, vertebral artery

## Abstract

**Objective:** Posterior C1–C2 instrumentation is technically demanding because of the complex osseous and vascular anatomy of the atlantoaxial region. This retrospective single-center case series aimed to describe the technical feasibility and observed screw-placement outcomes of a combined microscope-integrated augmented reality (AR)-assisted navigation workflow in patients with atlantoaxial instability. **Methods:** Two neurosurgeons and one radiologist assessed screw placement accuracy on computed tomography (CT) images using the Gertzbein–Robbins classification, applying the Bredow modification for the cervical spine. Cortical breaches were classified as Grade 1, <1 mm; Grade 2, 1 to <2 mm; Grade 3, 2 to <3 mm; Grade 4, 3 to <4 mm; and Grade 5, ≥4 mm, or as transverse foramen obstruction exceeding half the screw diameter. Grades 1 and 2 were considered acceptable for screw insertion. **Results:** A total of 40 screws were successfully placed in 10 patients, comprising 20 C1 lateral mass screws and 20 C2 pedicle screws, achieving an overall acceptable placement rate of 97.5% (39/40; 95% CI, 86.8–99.9%). All 20 C1 lateral mass screws were accurately positioned, while the acceptable placement rate for C2 pedicle screws was 95.0%. One C2 pedicle screw in a 1.7 mm pedicle was graded as Grade 3. No clinically apparent vascular or neurological injuries were observed. **Conclusions:** The combined microscope-integrated AR-assisted navigation workflow was feasible in this small case series, with a high observed acceptable screw-placement rate and no observed neurovascular complications. The independent contribution of AR and its effects on outcomes relative to other guidance techniques require evaluation in larger comparative studies.

## 1. Introduction

Atlantoaxial instability (AAI) may arise from a variety of etiologies, including congenital malformations, systemic inflammatory diseases, neoplastic processes, degeneration, and traumatic injuries to the spine. Stabilization of the C1–C2 vertebrae presents a significant obstacle due to their intricate anatomical configuration. Despite the continuous development and adaptation of surgical approaches, effective stabilization of the C1–C2 vertebrae remains an ongoing challenge. The complications most frequently encountered during fixation surgeries are the misplacement of screws and injuries to the vertebral artery (VA) [1,2,3,4,5].

Given the intricate neurovascular configurations and anatomical variability of the atlantoaxial segments, techniques for atlantoaxial instrumentation and imaging methodologies have undergone significant evolution. The aim of this evolution is to ensure a safe and effective approach to fusion [6]. The use of intraoperative fluoroscopy for instrumentation is well-accepted. However, the distortions resulting from cervical pathology may render conventional fluoroscopy insufficient to guarantee the safety and precision of screw placement. To mitigate the risk of life-threatening complications, a range of image-guided systems (IGSs) has been utilized during surgical procedures [7]. Over the past decade, the application of augmented reality (AR) techniques in spine surgery has become increasingly prevalent. These techniques offer 3D virtual images that are superimposed onto the surgical field, rendering the navigational procedures during surgery more intuitive and reducing the need for divided attention. Numerous studies and reviews have delved into the advantages and disadvantages of this innovative technique [8,9].

This retrospective single-center case series describes a combined navigation workflow incorporating preoperative computed tomography angiography, intraoperative computed tomography, image fusion, conventional navigation, microscope-integrated AR visualization, and fluoroscopic confirmation for posterior C1–C2 instrumentation. The study aimed to describe the technical feasibility of this workflow, the observed screw-placement outcomes, and the perioperative and follow-up complications. Because all patients underwent the same workflow without a comparator group, the study was not designed to determine the independent contribution of AR or its superiority over other guidance techniques.

## 2. Methods

### 2.1. Patient Selection

**Figure 1:**
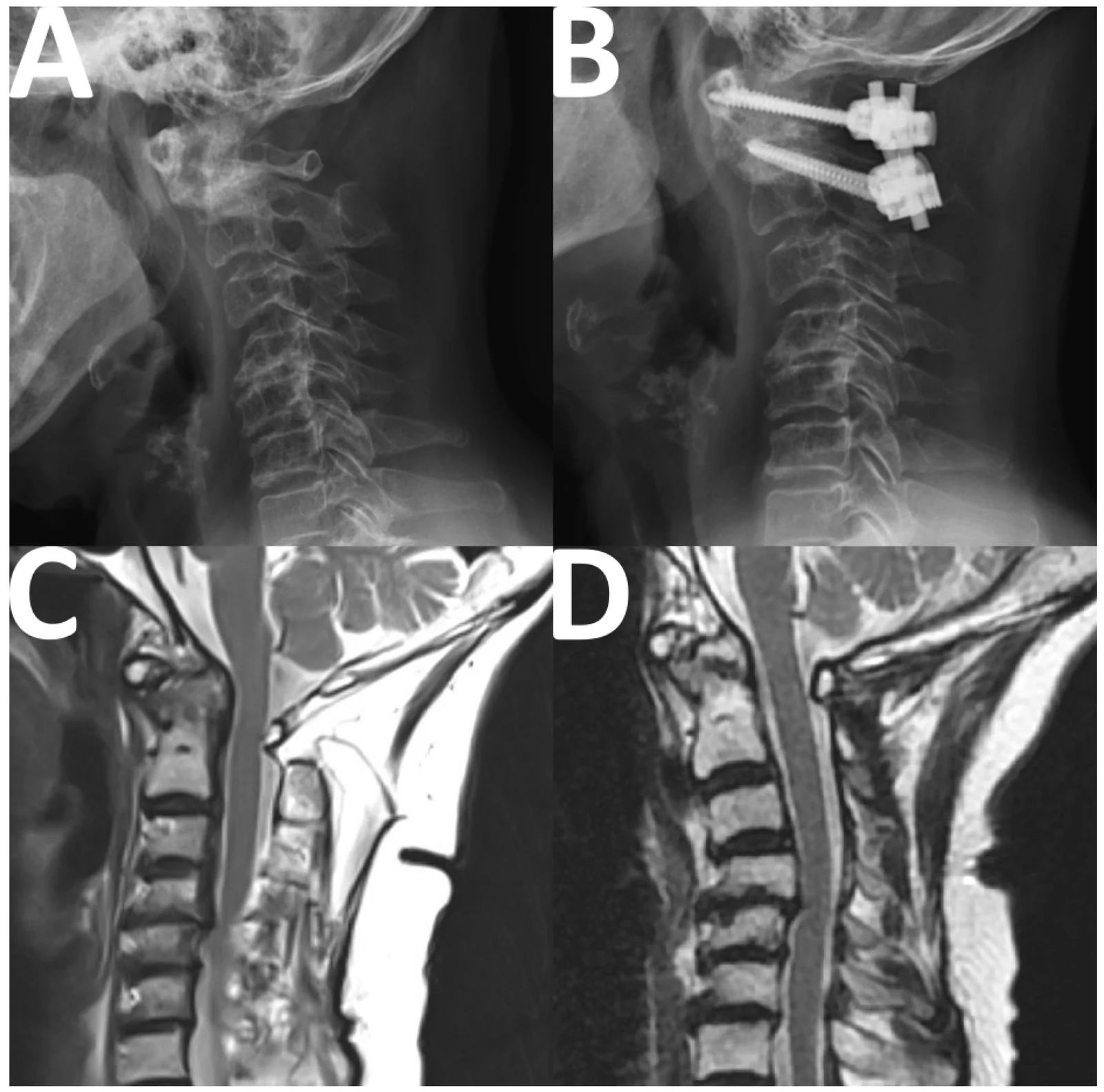
Atlantoaxial instability (AAI) in Case 10: (**A**) Flexion cervical spine radiograph demonstrates marked atlantoaxial instability. (**B**) Flexion radiograph obtained 6 months after surgery shows maintained reduction in the atlantoaxial subluxation following instrumentation. (**C**) Preoperative magnetic resonance imaging (MRI) reveals spinal canal compromise and spinal cord compression at the C1 level. (**D**) Postoperative MRI shows satisfactory reduction following C1–C2 instrumentation.

Between November 2021 and March 2025, 11 consecutive patients with symptomatic AAI underwent surgical treatment by the same neurosurgeon at a single hospital. This study was approved on 6 January 2025 by the Taichung Veterans General Hospital Institutional Review Board (No. CE24549A) and was performed in accordance with the ethical standards as stated in the 1964 Declaration of Helsinki and its later amendments. This case series has been reported in line with the PROCESS 2025 guideline [10]. The inclusion criteria were symptomatic AAI and AR-assisted surgical treatment by the study surgeon during this period. One patient with symptomatic os odontoideum, included in this cohort, was previously described in a case report by our group [11]. The present series expands upon that report by including additional patients, evaluating a larger number of screws, and providing longer postoperative follow-up. All 11 patients underwent AR-assisted surgery as part of the combined workflow described in Section 2.2; no patient was treated with conventional navigation or fluoroscopy alone, without AR. One patient was excluded because of concomitant anterior cervical discectomy and fusion, leaving 10 consecutive eligible patients in the final cohort; no other eligible patients were excluded. All 10 included patients underwent C1 lateral mass and C2 pedicle screw instrumentation using the same AR-assisted navigation system (Figure 1), with no alternative atlantoaxial fixation strategy used in the included cohort. AR-assisted navigation was used routinely for C1–C2 procedures performed by the study surgeon throughout the study period. No registration failures or instances of unreliable AR projection occurred, and no procedure required conversion to fluoroscopy or conventional navigation alone. Table 1 and Table 2 show the demographic data of the 10 patients.

### 2.2. Navigation and Augmented Reality Workflow

**Figure 2:**
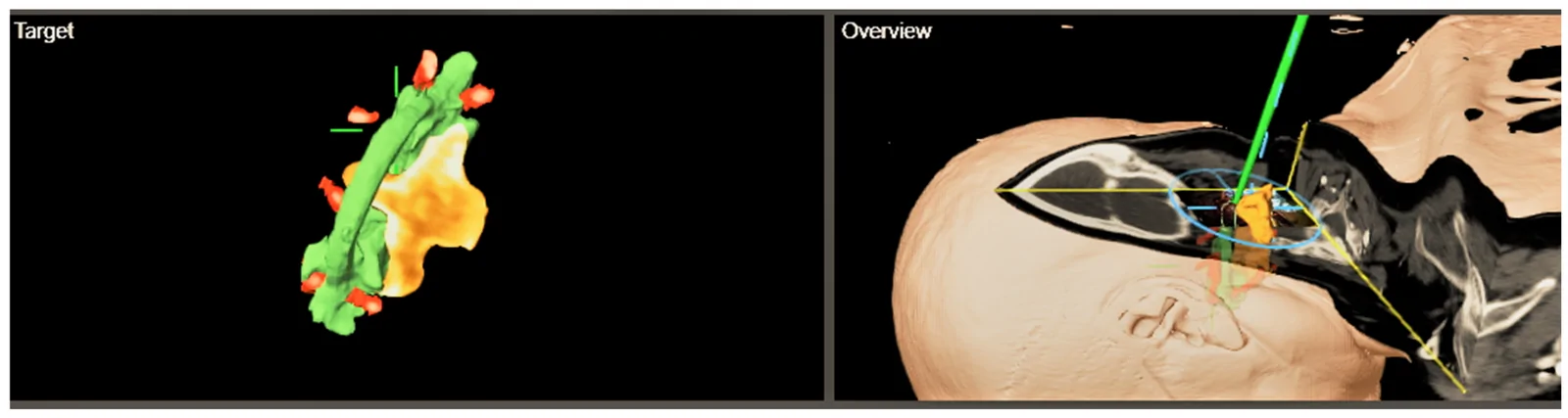
Three-dimensional reconstruction and intraoperative AR guidance. (**Left**) 3D reconstruction from preoperative CTA showing bilateral vertebral arteries (red) and bony anatomy (green/yellow). (**Right**) Intraoperative AR view illustrating real-time navigation during cervical spine surgery.

Patients were positioned prone with the head secured in a radiolucent skull clamp (DORO, Black Forest Medical Group, Freiburg im Breisgau, Germany), to which the patient reference array was attached. After exposure of the occiput, C1 posterior arch, and C2 laminae and isthmus, a registration matrix (Brainlab AG, Munich, Germany) was placed in the surgical field. An intraoperative three-dimensional scan was then acquired using either a sliding-gantry CT scanner (SOMATOM, Siemens Healthineers, Erlangen, Germany) or the cone-beam CT capability of an angiography system (ARTIS, Siemens Healthineers, Erlangen, Germany). Images were reconstructed at a slice thickness of 1.0–1.5 mm using a bone reconstruction kernel. The intraoperative dataset was automatically registered to the patient reference array using a rigid transformation (Automatic Image Registration, Curve, Brainlab). Registration was accepted only when the discrepancy between the physical pointer tip and its corresponding image location was ≤1 mm at the C2 spinous process, both C2 laminae, and the C1 posterior tubercle; otherwise, repeat scanning was required. Preoperative CTA was rigidly fused with the intraoperative dataset using a region of interest centered on C2; no deformable registration was used. The vertebral arteries (VAs) were segmented from the CTA images (Figure 2), and C1 and C2 were segmented from the intraoperative images using Elements Image Fusion and Elements Smartbrush (Brainlab AG, Munich, Germany). Screw trajectories were planned on the intraoperative dataset.

The operating microscope (KINEVO 900, software version 1.8, Carl Zeiss Meditec AG, Oberkochen, Germany) was tracked by the navigation camera and transmitted its magnification and focus settings to the navigation system, allowing the head-up display overlay to update with changes in microscope position, zoom, or focus. Individual segmented structures were selected for display as contours at the focal plane or as three-dimensional outlines, together with the planned screw entry points and trajectories. The overlay guided the extent of exposure, screw entry point selection, and initial drilling direction, while displaying the image-derived VA position at the focal depth. Alignment between the overlay and the exposed bone was monitored continuously as a check of registration. If the overlay shifted under drilling load, pressure on the instrument was released, and alignment was reconfirmed. After drilling, each screw tract was verified using the navigation pointer and monitor before screw insertion. A further intraoperative CT scan was obtained after instrumentation.

### 2.3. Surgical Techniques

For C1–C2 fixation, we used the AR-assisted navigation system to determine the screw entry points and trajectories, adopting a standard posterior midline technique. The procedure began with a posterior midline approach, where we dissected the muscle and soft tissues to expose the posterior elements of the cervical spine. The accuracy of the AR-assisted navigation system was verified at each stage by aligning the probe with anatomical landmarks.

**Figure 3:**
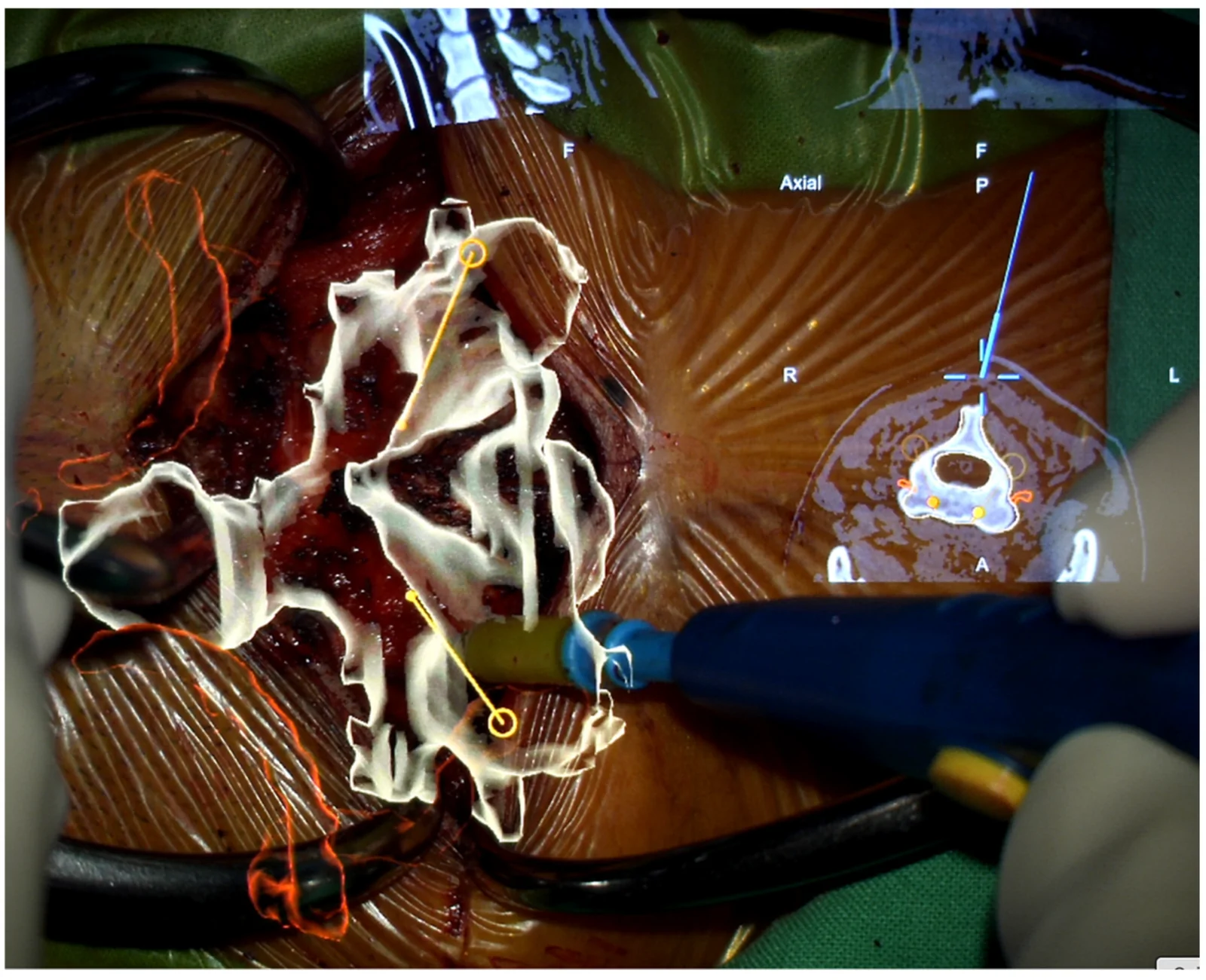
AR projection visualized under the microscope. The vertebral arteries are highlighted in red, and the planned screw entry points are indicated. Vascular and osseous structures are color-coded to aid intraoperative orientation. The crosshair in the axial navigation view corresponds to the focal point within the microscopic surgical field. This intraoperative image is from a patient other than the one described in our previous case report [11].

First, we exposed the lateral facets of the C1 posterior arch to identify the bilateral lateral masses. We then proceeded to uncover the spinous process, laminae, and the medial segments of the C2 lateral masses. Using AR technology, the bilateral VAs were coded in red and clearly visualized under the microscope (Figure 3). In addition, projections of the additional osseous structures were overlaid onto the surgical field (Figure 3). The navigation images displayed in the microscope eyepieces were also shown simultaneously on an external monitor (Figure 3).

Next, we utilized real-time navigation integrated with the AR system in the microscope to plan the entry points and trajectories for the C2 pedicle screws (Figure 3). By adjusting the magnification and zoom, the surgeon was able to visualize the anatomical structures without any obstruction from the surgical instruments. The accuracy of the planned trajectories was confirmed using AR-assisted navigation and intraoperative fluoroscopy.

A pilot hole was then created with a 2 mm high-speed burr. Before screw insertion, each drilled tract was checked by probing and using the navigation pointer and monitor to assess its alignment with the planned trajectory. After instrumentation, intraoperative CT was obtained to evaluate screw position. The subsequent surgical phases, including the application of rods, with or without C1 laminectomy, were carried out following standard protocols.

### 2.4. Radiological Evaluation

Final screw placement was evaluated on postoperative CT images acquired with a Spectral CT 7500 or IQon Spectral CT scanner (Philips Healthcare, Best, The Netherlands) or a Revolution CT scanner (GE HealthCare, Chicago, IL, USA) at 120–140 kVp with automatic tube current modulation. Only 3 mm reconstructions generated with a bone kernel were available for these postoperative studies, and axial and sagittal reformations were generated from these images for assessment of screw position. These acquisitions were separate from the intraoperative scans described in Section 2.2, which were reconstructed at 1.0–1.5 mm. Two neurosurgeons jointly reviewed the images and determined the grades by consensus. Both were aware of the operative details. A radiologist, blinded to the operative details, subsequently reviewed the images and confirmed the final classifications. Measurements were obtained from the available multiplanar reconstructions. Screw placement was classified using a cervical grading system based on Bredow et al. [12]. The following mutually exclusive breach intervals were applied: Grade 1, no breach or a breach of <1 mm; Grade 2, ≥1 to <2 mm; Grade 3, ≥2 to <3 mm; Grade 4, ≥3 to <4 mm; and Grade 5, ≥4 mm. Any screw causing transverse foramen obstruction exceeding half its diameter was classified as Grade 5, regardless of breach distance. For C2 pedicle screws, the osseous corridor was defined by the cortical margins of the C2 pedicle, and the breach distance was measured from the outer cortical margin of the perforated pedicle wall to the outermost aspect of the screw. For C1 lateral mass screws, where no pedicle is traversed, the corridor was defined by the cortical margins of the C1 lateral mass; the same breach intervals were applied, measured from the perforated cortical margin. The transverse foramen rule was applied at both levels. Grades 1 and 2 were considered acceptable, and the acceptable placement rate was calculated separately for C1 and C2 as the proportion of assessed screws meeting these criteria. The atlantodental interval (ADI) was measured on flexion lateral cervical radiographs acquired under the same conditions preoperatively and postoperatively. All ADI measurements were performed by a radiologist. The reliability of the measurements was not assessed.

### 2.5. Follow-Up and Complication Assessment

Postoperative radiographic follow-up comprised cervical radiographs obtained 2 days, 3–4 weeks, 3 months, 6 months, and 12 months after surgery, and a cervical CT scan obtained 1 month after surgery. Clinical follow-up continued thereafter at outpatient visits. The following complications were recorded: surgical site infection, cerebrospinal fluid leakage, screw loosening or breakage, loss of reduction, reoperation, readmission, and death.

### 2.6. Statistical Analysis

Continuous variables are presented as mean ± standard deviation, and categorical variables as numbers and percentages. For the main proportions, two-sided exact (Clopper–Pearson) 95% confidence intervals (CIs) were calculated. Screw placement was analyzed primarily at the screw level; because four screws were placed in each patient and screws within the same patient are not independent observations, placement was also summarized at the patient level, defined as all four screws in a patient being graded as acceptable (Grades 1–2). Given the small sample size, the analyses were descriptive, and no hypothesis testing was performed. The paired change in ADI was calculated for each patient and summarized as mean ± SD with a 95% CI based on the t distribution.

## 3. Results

A total of 40 screws were placed across 10 patients, including 20 C1 lateral mass screws and 20 C2 pedicle screws. The demographic characteristics of the patient cohort are summarized in Table 1. The mean patient age was 58.2 ± 22.9 years (range: 29–89), with four patients (40.0%) being male. The most common underlying pathologies were degenerative osteoarthritis (n = 3); symptomatic os odontoideum (n = 2); odontoid fracture (n = 1); and autoimmune-related diseases, including rheumatoid arthritis (n = 2), axial spondyloarthritis (n = 1), and systemic lupus erythematosus (n = 1). The mean operative time, defined as the time from skin incision to wound closure, was 349.1 ± 34.9 min, and the mean estimated blood loss was 480 ± 352.1 mL. Both values refer to the entire operation, including intraoperative imaging, registration, image fusion, AR setup, instrumentation, and any additional procedures such as C1 laminectomy. The mean length of hospital stay was 6.7 ± 1.3 days. The mean clinical follow-up period was 31.1 ± 12.0 months, with a minimum follow-up duration of 12 months.

The mean ADI was 8.3 ± 3.6 mm preoperatively and 2.4 ± 0.4 mm postoperatively (Table 2). The mean paired reduction was 5.9 ± 3.5 mm (95% CI, 3.5–8.4 mm), and the ADI decreased in all 10 patients (range of reduction, 1.7–12.3 mm). The average C2 pedicle width was 4.8 ± 2.5 mm on the right and 5.7 ± 2.0 mm on the left, with individual values ranging from 1.7 to 10.6 mm (Table 2).

Screw placement accuracy was assessed using a grading system based on the extent of cortical breach. Among the C1 lateral mass screws, 17 screws (85%) were classified as Grade 1 (without breach or breach < 1 mm), and three screws (15%) as Grade 2 (cortical breach 1 to < 2 mm), resulting in an acceptable placement rate of 100.0% (20/20; 95% CI, 83.2–100%). Among the C2 pedicle screws, 17 (85%) were classified as Grade 1, two (10%) as Grade 2, and one (5%) as Grade 3 (cortical breach 2 to <3 mm). No screws were classified as Grade 4 or 5. The acceptable placement rate for C2 pedicle screws was therefore 95.0% (19/20; 95% CI, 75.1–99.9%), and the overall acceptable placement rate was 97.5% (39/40; 95% CI, 86.8–99.9%) (Table 3). At the patient level, all four screws were graded as acceptable in 9 of 10 patients (90.0%; 95% CI, 55.5–99.7%).

The distribution of non-Grade 1 screws by patient is shown in Table 2, and the characteristics of individual screws are detailed in Supplementary Table S1. The five Grade 2 screws occurred in three patients aged 29, 45, and 73 years, and the single Grade 3 screw occurred in a 70-year-old patient with a 1.7 mm C2 pedicle. All screws in the three oldest patients (81, 81, and 89 years) were graded as Grade 1. No apparent association between age and screw placement accuracy was observed; however, formal analysis was not feasible given the small number of patients and non-Grade 1 screws.

Five screws were classified as Grade 2 and one as Grade 3. These screws were retained following intraoperative assessment of screw position and fixation stability. No clinically apparent vascular or neurological complications were observed during the reported follow-up period (0/10 patients; 95% CI, 0–30.8%). No surgical site infection, cerebrospinal fluid leakage, screw loosening or breakage, loss of reduction, reoperation, readmission, or death occurred, and no patients were lost to follow-up.

## 4. Discussion

**Figure 4:**
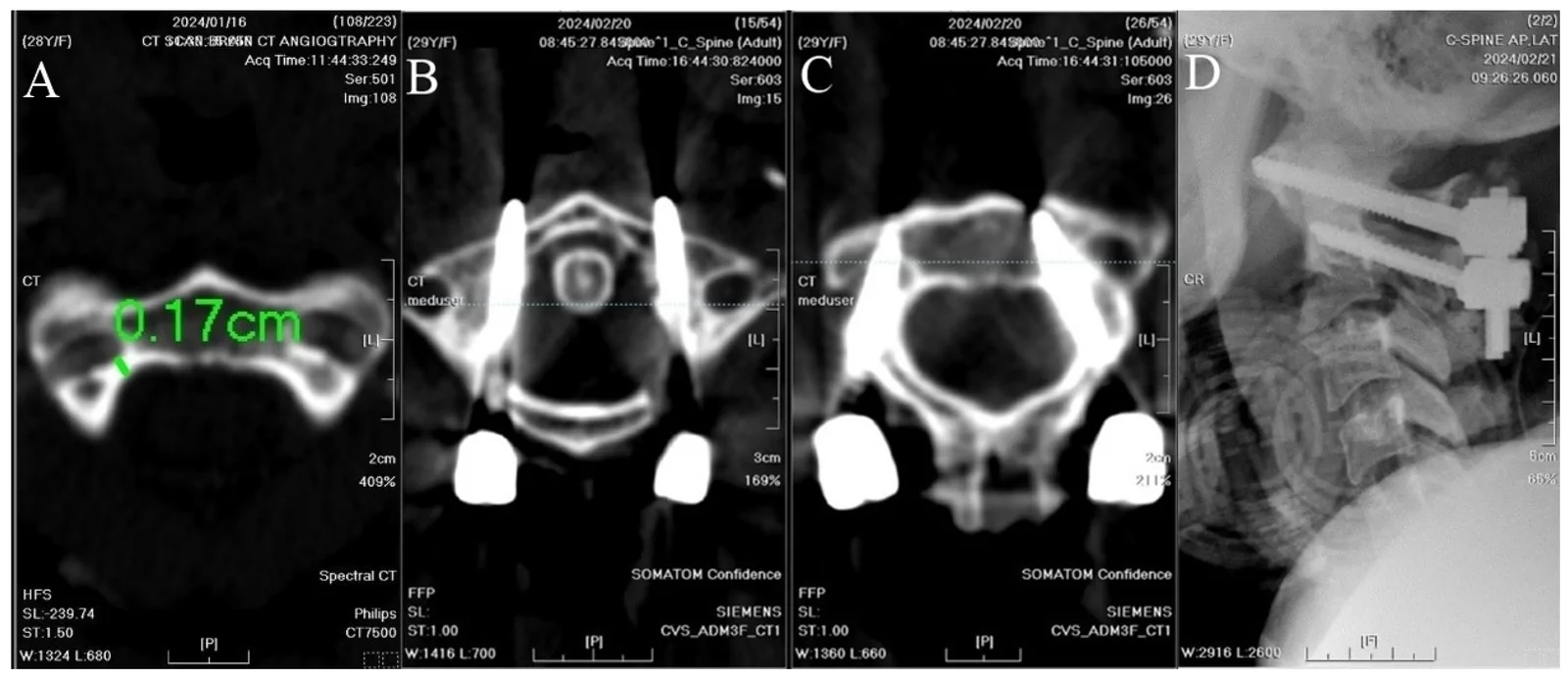
Preoperative and postoperative images: (**A**) An axial CT image indicated a narrow right C2 pedicle with a diameter of 1.7 mm. (**B**) Postoperative CT showed precise C1 lateral mass screw placement. (**C**) A lateral breach was noted at the right C2 pedicle screw site without any vascular or neural injury. (**D**) Lateral view of postoperative radiograph follow-up.

In this retrospective series of 10 patients, posterior C1–C2 instrumentation was performed using an AR-assisted navigation workflow. The observed acceptable screw-placement rate was 97.5% overall, comprising 100% for C1 lateral mass screws and 95.0% for C2 pedicle screws. One C2 pedicle screw was graded as Grade 3. This screw was placed in the narrowest pedicle in the cohort (1.7 mm; Figure 4A,C), and a breach occurred despite the use of the combined navigation workflow. No vascular or neurological complications were observed during follow-up. These descriptive findings support the technical feasibility of the workflow in this setting; however, the small sample size and absence of a comparator group preclude establishing its safety profile or determining whether AR improved screw placement or clinical outcomes.

Real-time image-guided navigation has been associated with a reduced incidence of implant-related neural and vascular injury in craniovertebral junction surgery [13]. With advancements in optical and electronic technologies, AR-assisted navigation systems have shown promising results in spinal surgeries [8,9]. For cervical procedures, microscope-integrated AR has been reported mainly in anterior approaches [14] or in mixed spinal cohorts [15], with atlantoaxial application limited to an isolated case report [11]. Mixed reality delivered through a head-mounted display has been evaluated for C1–C2 pedicle screw placement, but only in three-dimensional printed models, not in patients [16]. Previous reports have described the use of AR-assisted navigation for C1–C2 instrumentation in patients with AAI [11,17]. These platforms differ in registration workflow, display modality, and line-of-sight requirements, and evidence obtained with one platform does not necessarily transfer to another. Table 4 summarizes the guidance technologies cited in this manuscript by category, representative studies, study setting, and anatomical region.

Despite the advantages, some surgeons may question the ergonomics of maneuvering drilling tools and screwdrivers while looking through a microscope, especially considering the short working distance above the surgical field. In fact, with appropriate focus length and magnification, AR holography projected onto the surgical field vividly visualizes the C1–C2 bony anatomy and VAs under the microscope. This allows for real-time insight into the spatial relationship between the screw entry point and the VA path, bolstering surgeon confidence during the procedure. During pilot hole drilling, with proper focus length, surgeons can perform the bony work similarly to intracranial microsurgery using a microscope, without the potential issue of intraoperative navigation deviation caused by structural contraction after cerebrospinal fluid drainage.

Avoiding neurovascular injuries in C1–C2 surgeries presents a significant challenge, particularly in the cervical spine, where pedicles are notably smaller, narrower, and exhibit greater variability than those in the thoracic or lumbar regions. This complexity is further heightened by the intricate neurovascular structures within the atlantoaxial region, adding to the technical demands of C1–C2 procedures. Anatomical variations, such as a high-riding vertebral artery (VA)—defined by an isthmus height of less than 5 mm and/or an internal height of less than 2 mm on the sagittal CT section transecting the mid-portion of the C1–C2 facet joint—or a narrow C2 pedicle, defined as a pedicle width of 4 mm or less [31], significantly increase the risk associated with C2 pedicle screw insertion. Even in patients without such variations, previous studies have reported an average C2 pedicle width of 5.5 ± 1.1 mm [32] and a safety margin of 6.9 ± 1.5 mm for C1 lateral mass screw fixation [33]. These findings highlight the importance of precise screw placement to avoid vascular or neural injury.

The reported incidence of VA injury varies with surgical technique, ranging from 1.2% to 13.1% per screw for C1–C2 transarticular screws [34] and from 2.1% to 5% for C1 lateral mass and C2 pedicle screws [35,36,37]. A C1 lateral mass–C2 pedicle screw–rod construct was used in all patients in this series. In the patient with the narrowest pedicle, intraoperative reduction was required (ADI 8.5 mm preoperatively, 2.4 mm postoperatively; Table 2), and the screw–rod construct was selected to permit manipulation of alignment and maintain the corrected position, whereas transarticular screw placement generally requires satisfactory reduction beforehand [38]. This consideration does not establish the superiority of pedicle fixation over C2 pars or translaminar screws, which remain alternatives when the pedicle is narrow. In a finite element study, Lu et al. found that C2 isthmus screw fixation provided stability comparable to that of C2 pedicle screw fixation in a model of atlantoaxial dislocation, with sufficient pullout strength, although these findings were derived from a single healthy-volunteer model and await clinical validation [39]. The right C2 pedicle measured 1.7 mm (Figure 4A), and the screw used was 3.5 mm in diameter and 28 mm in length—3.5 mm being the smallest diameter available at our institution. Preoperative CTA showed no high-riding VA or other vascular variation and approximately 2 mm of separation between the VA and the adjacent bony wall of the transverse foramen, which was considered during trajectory planning; this preoperative measurement does not, however, establish the final screw-to-artery distance. Postoperative CT demonstrated a Grade 3 lateral cortical breach (2 mm from the outer cortical margin of the lateral pedicle wall to the outermost aspect of the screw) with approximately 1 mm of encroachment into the transverse foramen measured from its medial wall, corresponding to about 29% of the screw diameter and thus below the Grade 5 threshold. No screw redirection was required, and no intraoperative bleeding or clinically apparent postoperative neurovascular complications occurred. Because the patient remained asymptomatic, postoperative imaging was limited to cervical CT; CT and clinical observation alone cannot exclude a clinically silent vascular injury [3]. This case illustrates the anatomical limitations of navigated screw placement in an exceptionally narrow pedicle—the workflow could define the available corridor but not enlarge it—and the uneventful course should not be taken as confirmation of the safety of the selected trajectory.

Although screw placement accuracy was the primary focus of this study, durable C1–C2 fusion is the ultimate goal of posterior atlantoaxial instrumentation. Montemurro et al. evaluated 74 patients aged ≥65 years who underwent surgery for C1 or C2 fractures and assessed fusion on CT at 1 year using the Lenke grading system; good fusion (Lenke grades A–B) was achieved in 71.6% of patients overall, 79.5% of those treated through a posterior approach, and 80.0% of those treated with the Harms technique [40]. In a case series and literature review of posterior C1 lateral mass–C2 pedicle/pars screw–rod fixation, Unni et al. noted that fusion rates of up to 100% have been reported with the Goel–Harms technique, compared with nonunion rates of up to 80% with earlier wiring techniques [41]. Because fusion was not formally assessed in the present series, our results cannot be compared with these rates, and whether AR-assisted screw placement influences fusion remains to be determined.

The advancement of intraoperative navigation technologies has markedly improved the precision and efficiency of screw placement during spine surgeries, in addition to enhancing the accuracy of intraoperative anatomical localization [42,43]. Given the potential discrepancies between preoperative CT imaging and the actual intraoperative surgical anatomy and alignment [18], the use of cone-beam CT- or O-arm-based intraoperative navigation has emerged as a feasible solution [20,21,22]. Recent studies have reported overall breach rates ranging from 2.9% to 11.1% [23,24,25,26]. Gan et al. reported a breach rate of 16.7%, with 7 of 42 C2 pedicle screws showing breaches, most of which were minor and did not exceed 2 mm [27].

In contrast to the microscope-based AR system, conventional navigation systems often face technical limitations due to line-of-sight challenges. These occur when optical obstructions prevent the surgical tool’s tracing marker from being captured by the external camera, leading to disruptions in navigation [44]. One possible solution to this problem is to automate the calibration between the reference array on the microscope and another reference attached to the skull clamp, which is placed outside the surgical field. This method may help maintain intraoperative accuracy and also reduce issues related to infrared sensor blockage. Furthermore, the microscope-based AR system allows surgeons to maintain their focus without needing to look at a separate monitor. By integrating all navigation data directly into the microscope, it seamlessly merges with the anatomical views of the surgical area. This integration helps minimize potential distractions for the surgeon. However, surgeons must be cautious to ensure that the AR projections accurately align with the actual anatomy.

A significant challenge in surgical navigation arises from the potential discrepancy between the anatomical positioning of a patient during surgery (prone) and their positioning during preoperative CT scans (supine). This difference in positioning can result in navigation inaccuracies, which increase the risk of intraoperative complications. When a patient is scanned in a supine position and then operated on in a prone position, the anatomical landmarks used for navigation can shift, leading to potential errors in the surgical process [18]. To address and mitigate these issues, we fused preoperative CTA images with intraoperative CT images. This integration allows for more precise alignment of anatomical structures. By processing both sets of images within the navigation software, we could cross-verify anatomical alignment, ensuring that the navigation system accurately reflected the patient’s anatomy during surgery. This approach allows surgeons to concentrate on specific cervical spinal segments with increased confidence and accuracy. This integrated imaging workflow was intended to account for positional differences between preoperative and intraoperative imaging and to support anatomical orientation during C1–C2 instrumentation.

This series included only open procedures and, therefore, does not establish the feasibility or safety of AR-assisted navigation for percutaneous C1–C2 fixation. Building on previous reports of minimally invasive cervical fixation [45,46,47], future studies could evaluate the potential application of this combined navigation workflow to percutaneous C1–C2 instrumentation.

### Limitations of the Study

The principal limitations of this study are its retrospective design, small sample size, single-center and single-surgeon setting, and absence of a comparator group. The small number of patients and non-Grade 1 screws also precluded analysis of whether age or the severity of degenerative changes influenced navigation or screw placement accuracy. Preoperative CTA, intraoperative CT, image fusion, conventional navigation, AR visualization, fluoroscopic confirmation, and surgeon experience may all have contributed to the observed screw-placement results. Accordingly, this study cannot determine the independent effect of AR on accuracy, safety, or clinical outcomes. Furthermore, the absence of observed neurovascular complications in 10 patients is insufficient to establish a safety profile (upper 95% confidence limit, 30.8%). Larger comparative studies are needed to evaluate the additional contribution of AR to established navigation workflows. The operative time and blood loss reported here refer to the entire operation, and the time required specifically for intraoperative CT, registration, image fusion, and AR setup was not recorded; therefore, the workflow-specific time burden could not be quantified.

Regarding radiological evaluation, CT measurement planes were not consistently confirmed to be perpendicular to the screw axis, which may have affected breach measurements and grading. Only 3 mm reconstructions were available for the postoperative studies, so reformatting along the screw axis and re-measurement were not feasible. This is most consequential for the single Grade 3 screw, whose breach was measured at 2.0 mm, exactly at the Grade 2/3 boundary; a difference of less than 1 mm would reclassify this screw as Grade 2, and the acceptable placement rate would then be 40/40 (100%) rather than 39/40 (97.5%). Screw placement was graded by consensus between two neurosurgeons who were aware of the operative details. Although a radiologist blinded to the operative details subsequently confirmed the grades, independent interobserver agreement could not be assessed, and observer bias cannot be excluded. Similarly, ADI was measured by a single radiologist, and the intra- and interobserver reliability of these measurements was not evaluated. Regarding follow-up, bony fusion was not formally assessed, and pseudarthrosis was therefore not evaluated. Although no screw loosening or breakage, loss of reduction, or reoperation was observed on serial radiographs, the reduction measured postoperatively does not by itself establish durable stabilization. Furthermore, standardized neurological, pain, myelopathy, and functional outcome measures were not systematically collected; therefore, the clinical efficacy of the procedure could not be evaluated.

While the innovative technique of integrating AR-assisted navigation in surgical procedures offers numerous advantages, it also presents inherent limitations. One of the primary challenges is the system’s reliance on the surgeon’s proficiency and familiarity with AR technologies, particularly for those who have not regularly used a microscope. Implementing comprehensive training modules tailored to surgeons, assisting nurses, and radiological technicians requires significant time and financial resources.

Another critical consideration is the radiation exposure of patients. Navigation with intraoperative 3D imaging can reduce intraprocedural fluoroscopy and therefore occupational exposure [29], although total patient dose depends on the number of three-dimensional acquisitions performed [48]. Our protocol required a preoperative CTA and at least one intraoperative 3D acquisition, with a further post-instrumentation scan. Reductions in effective dose have been reported for projector-based AR-assisted navigation systems with integrated cone-beam CT [29]; however, those imaging chains differ from ours. Radiation dose was not quantified in this series, and the dose implications of the workflow described here therefore remain undetermined.

Finally, the substantial capital expenditure required for setup, along with ongoing maintenance costs, reflects the financial constraints of adopting this emerging technology.

## 5. Conclusions

An AR-assisted navigation workflow was feasible for posterior C1–C2 instrumentation in this retrospective single-center series of 10 patients, with a high observed acceptable screw-placement rate and no observed vascular or neurological complications. These descriptive findings do not establish the independent benefits or safety profile of AR. Larger comparative studies are required to determine whether adding AR to conventional navigation improves screw placement or clinical outcomes.

## Figures and Tables

**Table 1:** Demographic data of the patients.

Characteristics	Value
Total number of patients	10
Age, years, mean ± SD (range)	58.2 ± 22.9 (29–89)
Male, n (%)	4 (40.0)
Diagnosis, n (%)	
Degenerative osteoarthritis	3 (30.0)
Symptomatic os odontoideum	2 (20.0)
Odontoid fracture	1 (10.0)
Rheumatoid arthritis	2 (20.0)
Axial spondyloarthritis	1 (10.0)
Systemic lupus erythematosus	1 (10.0)
Operative time, min, mean ± SD *	349.1 ± 34.9
Estimated blood loss, mean ± SD *	480.0 ± 352.1
Length of stay, days, mean ± SD	6.7 ± 1.3
Follow-up, months, mean ± SD (range)	31.1 ± 12.0 (12–51)

SD, standard deviation. * Operative time and estimated blood loss refer to the entire operation; the duration of individual phases was not recorded.

**Table 2:** Data for instrumentation surgeries.

Case No.	Age (Years)	Preoperative ADI (mm)	Postoperative ADI (mm)	C2 Pedicle Width (mm) (Right)	C2 Pedicle Width (mm) (Left)	Non-Grade 1 Screws
1	37	4.0	1.5	5.8	6.3	—
2	89	4.0	2.3	3.9	3.0	—
3	31	12.2	2.1	3.2	3.4	—
4	46	11.1	2.4	4.7	7.5	—
5	81	7.8	2.8	10.6	9.2	—
6	45	7.8	2.1	4.4	6.1	Grade 2 × 1
7	81	5.7	2.1	6.3	6.2	—
8	29	15.1	2.8	2.5	5.4	Grade 2 × 2
9	73	6.6	3.0	5.1	6.2	Grade 2 × 2
* 10	70	8.5	2.4	1.7	3.3	Grade 3 × 1
Mean ± SD	58.2 ± 22.9	8.3 ± 3.6	2.4 ± 0.4	4.8 ± 2.5	5.7 ± 2.0	—

Measurement methods: Atlantodental interval was measured on flexion cervical spine radiographs; C2 pedicle width was assessed on cervical spine computed tomography. ADI, atlantodental interval; SD, standard deviation. * Case 10 has a grade 3 breach at the right-side pedicle screw.

**Table 3:** Accuracy assessment of C1–C2 instrumentation.

	Grade 1	Grade 2	Grade 3	Grade 4	Grade 5	Acceptable Placement Rate (95% CI)
C1 lateral mass screws (total: 20 screws)	17	3	0	0	0	100.0% (83.2–100)
C2 pedicle screws (total: 20 screws)	17	2	1	0	0	95.0% (75.1–99.9)
Total (n = 40)	34	5	1	0	0	97.5% (86.8–99.9)

Screw placement was graded by cortical breach distance: Grade 1, <1 mm; Grade 2, 1 to <2 mm; Grade 3, 2 to <3 mm; Grade 4, 3 to <4 mm; and Grade 5, ≥4 mm. Transverse foramen obstruction exceeding half the screw diameter was classified as Grade 5, regardless of breach distance. CI, confidence interval (exact Clopper–Pearson method). Acceptable placement was defined as Grades 1–2.

**Table 4:** Image-guided and augmented reality platforms cited in this manuscript.

Category	Imaging and Registration	Visualization	Cited Studies (Setting, Region)
Conventional navigation	Preoperative CT; rigid reference array	Orthogonal reconstructions on monitor	Costa [18] (clinical); Itoh [19] (clinical, C1–C2)
Navigation with intraoperative 3D imaging	iCT/CBCT/O-arm; rigid reference array	Orthogonal reconstructions on monitor	Haemmerli [20], Czabanka [21], Smith [22], Ishikawa [23], Shimokawa [24], Chachan [25], Oearsakul [26], Gan [27] (clinical, cervical/C1–C2)
Video-based ARSN	Integrated CBCT in hybrid OR; adhesive skin markers; cameras in flat detector; no bony reference	3D anatomy and planned trajectory overlaid on live video on external monitor	Elmi-Terander [28] (cadaveric, thoracic); Edström [29] (clinical, thoracolumbar)
Microscope-integrated AR (present study)	iCT/CBCT + preoperative CTA fusion; rigid reference array; microscope tracked by navigation camera	Segmented contours and trajectories in microscope oculars, updating with zoom and focus	Carl [15] (clinical, mixed levels); Onuma [14] (clinical, anterior cervical); Li [11] (clinical, C1–C2 case report)
Head-mounted display AR	Preoperative or intraoperative CT; HMD-based tracking	Holographic overlay in HMD	Chang [30] (clinical, thoracolumbar)
Mixed reality	Preoperative CT-derived 3D model; three-point landmark registration; electromagnetic tracking; no intraoperative imaging	Holographic overlay with interaction via HMD (HoloLens)	Wu [16] (model, C1–C2)

ARSN, augmented reality surgical navigation; CBCT, cone-beam computed tomography; HMD, head-mounted display; iCT, intraoperative computed tomography.

## Data Availability

The data are not publicly available but may be obtained from the corresponding author upon reasonable request and with permission from Taichung Veterans General Hospital.

## Supplementary Materials

The following supporting information can be downloaded at: https://www.mdpi.com/article/10.3390/jcm15207812/s1, Table S1. Characteristics of the six non-Grade 1 screws.
