# Supplementary material for: Microscope-Integrated Augmented Reality-Assisted Navigation for C1–C2 Instrumentation: A Retrospective Single-Center Case Series
Source: J Clin Med. 2026 Oct 10;15(20):7812. doi: 10.3390/jcm15207812 (PMC13705249; doi:10.3390/jcm15207812)
Supplement: Supplementary file 1 [file jcm-15-07812-s001.zip › jcm-4480827-supplementary.pdf]

Supplement Table S1. Characteristics of the six non-Grade 1 screws.

Case No.	Level	Side	Grade	Breach distance (mm)	Direction
6	C1 lateral mass	Right	2	1.5	Medial
8	C1 lateral mass	Right	2	1.8	Medial
	C1 lateral mass	Left	2	1.5	Medial
9	C1 lateral mass	Right	2	1.5	Medial
	C2 pedicle	Right	2	1.5	Medial
10	C2 pedicle	Right	3	2.0	Lateral

Grade 2, cortical breach ⩾1 to <2 mm; Grade 3, cortical breach ⩾2 to <3 mm. Breach distance was measured at the point of maximum cortical breach on postoperative multiplanar CT reconstructions. All other 34 screws were graded as Grade 1.
